# Stimulation settings in subthalamic nucleus deep brain stimulation for parkinson’s disease – a retrospective single-center observational study

**DOI:** 10.1186/s42466-026-00477-5

**Published:** 2026-07-02

**Authors:** Charlotte Schedlich-Teufer, Gregor A. Brandt, Christina van der Linden, Hannah Jergas, Juan Carlos Baldermann, Vasilija Stopic, Gereon R. Fink, Veerle Visser-Vandewalle, Till A. Dembek, Michael T. Barbe, Jan Niklas Petry-Schmelzer

**Affiliations:** 1 Department of Neurology, Faculty of Medicine and University Hospital Cologne, University of Cologne, Cologne, Germany; 2 Department of Neurology With Experimental Neurology, Charité - Universitätsmedizin Berlin, corporate member of Freie Universität Berlin and Humboldt Universität Zu Berlin, Berlin, Germany; 3 Department of Psychiatry and Psychotherapy, Medical Center - University of Freiburg, Faculty of Medicine, University of Freiburg, Freiburg, Germany; 4 Cognitive Neuroscience, Institute for Neuroscience and Medicine (INM-3), Jülich Research Center, Jülich, Germany; 5 Department of Stereotactic and Functional Neurosurgery, Faculty of Medicine and University Hospital Cologne, University of Cologne, Cologne, Germany

**Keywords:** Parkinson’s disease, Deep brain stimulation, Subthalamic nucleus, Pulse width, Frequency, Directional, Interleaving, Bipolar

## Abstract

**Background:**

Deep brain stimulation (DBS) of the subthalamic nucleus (STN) is a well-established treatment for Parkinson’s disease (PD). Beyond basic omnidirectional, monopolar stimulation, advanced stimulation settings (AS), such as directional or vertical current steering, variation of pulse width and frequency, bipolar and interleaving stimulation are increasingly available. Our aim was to summarize current evidence on AS in STN-DBS in PD and systemically report their application and potential benefits in clinical routine.

**Methods:**

In this retrospective single-center observational study, we analyzed stimulation settings of 145 patients with bilateral STN-DBS 3, 6, and 12 months postoperatively. Secondary outcomes included preoperative levodopa response, lead positions, and postoperative reduction of levodopa-equivalent daily dose (LEDD) in patients staying with basic stimulation settings (BS) compared to those initially with AS at 3-months follow-up and those with a change from BS to AS.

**Results:**

AS were applied in 40.7%, 55.9%, and 73.8% of patients at 3, 6, and 12 months respectively. LEDD reduction after three months was higher in patients remaining with BS or initially AS than in patients with a switch to AS after three months, while there was no difference at 12 months. Median distance of leads to the center of gravity of the motor-STN was slightly larger when AS were applied.

**Conclusions:**

AS are frequently employed in clinical routine at a specialized DBS center. They may compensate for deviant lead placement in terms of stimulation efficacy measured by postoperative LEDD reduction. Prospective studies are warranted, focusing on specific AS indications in chronic DBS to optimize individual patient outcomes.

**Supplementary Information:**

The online version contains supplementary material available at 10.1186/s42466-026-00477-5.

## Background

Deep brain stimulation (DBS) of the subthalamic nucleus (STN) is a well-established treatment for patients with Parkinson’s disease (PD). While exact lead placement is crucial for patient benefit, optimized postoperative stimulation settings can potentially increase the therapeutic effect while avoiding stimulation-induced side effects to improve individual therapeutic benefit [1].

Recent technical developments allow for advanced stimulation settings (AS) in clinical routine, such as directional current steering [2–5], vertical current steering [6–8], variation of stimulation pulse widths [9–13] and frequencies [14–17], as well as bipolar [18, 19] and interleaving or independent frequency stimulation [20–22]. Due to this multitude of stimulation parameters, complexity of DBS programming has increased immensely. To date there is no comprehensive framework guiding clinicians on when to employ which AS. It remains elusive whether AS are mainly advantageous due to the avoidance of side effects or if they have unique symptom-related benefits.

This retrospective observational study quantifies the use of AS in clinical routine at a center specialized in the care of movement disorders and DBS. We analyzed whether the application of AS is related to preoperative levodopa response and lead positions and whether it impacts DBS outcomes. We further provide an overview of the current evidence for applying different stimulation settings in PD patients with STN-DBS.

## Methods

### Participants and ethical approval

We retrospectively analyzed the stimulation settings of PD patients treated with bilateral STN-DBS as per clinical routine at our center. Data was gathered from routine follow-ups 3, 6, and 12 months after surgery. Only patients implanted with DBS devices capable of all predefined advanced stimulation settings and complete follow-ups within the first postoperative year were included. We acquired data on age, sex, disease duration, PD motor subtype, Hoehn and Yahr stage, levodopa-equivalent daily dose (LEDD) [23], Unified Parkinson’s Disease Rating Scale-III (UPDRS-III) [24] in medication-ON and -OFF states at time of preoperative indication testing (levodopa response), and stimulation settings from our in-house patient database. Additionally, we calculated the charge per second (CPS = pulse width x amplitude x frequency) as a measure of the applied energy for each stimulation setting. Charge per second is a suitable parameter for comparing current-controlled stimulation between patients independent of impedances and reflects the energy applied in the surrounding tissue [25]. The study was carried out following the Declaration of Helsinki. Formal ethical approval for this retrospective analysis was waived by the local ethics committee (University of Cologne, Study No. 25–1167).

### Postoperative programming

DBS programming in the study cohort was performed during clinical routine. This routine included initiating stimulation approximately 5—7 days postoperatively at the contact level where the best intraoperative effect was recorded (defined by clinical testing in combination with electrophysiological measurements), with a standard frequency of 130 Hz and a standard pulse width of 60 µs. Patients were discharged home after initiation of stimulation. Careful amplitude and medication titration continued at an external neurorehabilitation center for several weeks. Three months postoperatively (3-months follow-up; 3-MFU), patients underwent an in-house monopolar contact review after overnight withdrawal of dopaminergic medication to determine the most efficient contact level again. Stimulation settings were chosen by an experienced DBS clinician with the aim to increase stimulation effect while minimizing clinically monitored stimulation induced side effects. Patients were usually allowed to adjust amplitudes via their handheld device within certain ranges. DBS settings and medication were then further adjusted at regular visits 6 and 12 months postoperatively.

For further analysis, basic stimulation settings (BS) were defined as omnidirectional single contact level stimulation at 60 µs and 130 Hz. AS were defined as directional current steering, vertical current steering, pulse width ≠ 60 µs, frequency ≠ 130 Hz, bipolar stimulation settings, and interleaving stimulation (including independent frequency stimulation). In directional and vertical current steering, individual contact selection and independent amplitude titrations per contact were possible.

### Lead reconstruction

Patients received preoperative MRI and postoperative CT scans as per clinical routine. DBS leads were localized using the Lead-DBS toolbox (www.lead-dbs.org) [26]. T1- and T2-weighted MRI scans were linearly co-registered using SPM 12 (http://www.fil.ion.ucl.ac.uk/spm/software/spm12/, n = 143) or Advanced Normalization Tools (ANTs, http://stnava.github.io/ANTs/, n = 2) [27]. MRI and CT images were co-registered linearly using ANTs [27] (n = 142) or FMRIB’s Linear Image Registration Tool (FLIRT, https://fsl.fmrib.ox.ac.uk/, n = 3) [28]. Co-registration results were corrected for brain shift with an automatized subcortical refinement module implemented in Lead-DBS [26]. MRI scans were normalized nonlinearly into the Montreal Neurological Institute (MNI) space (ICMB 2009b NLIN asymmetric) using the symmetric diffeomorphic registration approach (SyN) implemented in ANTs and the “subcortical refine” setting as implemented in Lead-DBS (n = 142). If ANTs did not provide sufficient results, we used three-step affine normalization ANTs [29] (n = 2) and in one case FMRIB’s Nonlinear Image Registration Tool (FNIRT, https://fsl.fmrib.ox.ac.uk/) [30]. Leads were pre-reconstructed with the PaCER algorithm (https://adhusch.github.io/PaCER/, n = 144) [31] and were manually refined. In one case we used TRAC/CORE algorithm [32] since PaCER algorithm failed. We visually controlled all steps for accuracy. Left hemispheric leads were nonlinearly flipped to the right hemisphere. STN dimensions and subdivisions were defined using the DBS Intrinsic Template (DISTAL) atlas [33]. For further analysis of lead positions, the distance from the center between the directional contact levels to the center of gravity of the motor STN in the DISTAL atlas was calculated [33].

### Statistical analysis

First, we analyzed the proportions of different stimulation settings during the regular follow-ups within the first postoperative year. We analyzed changes of the application of each different stimulation settings over time using a mixed-effects logistic regression model with subject-specific random intercepts and time as a fixed effect. Effect sizes for the trends in each setting over time are reported as estimates and odds ratios with corresponding false discovery rate (FDR) corrected p-values to account for multiple comparisons.

To examine differences in lead positions, the median distance to the center of gravity of the motor STN was compared between leads with BS at all follow-ups and leads with a switch to AS at least once within the follow-up period using a Mann–Whitney U test.

**Fig. 1:**
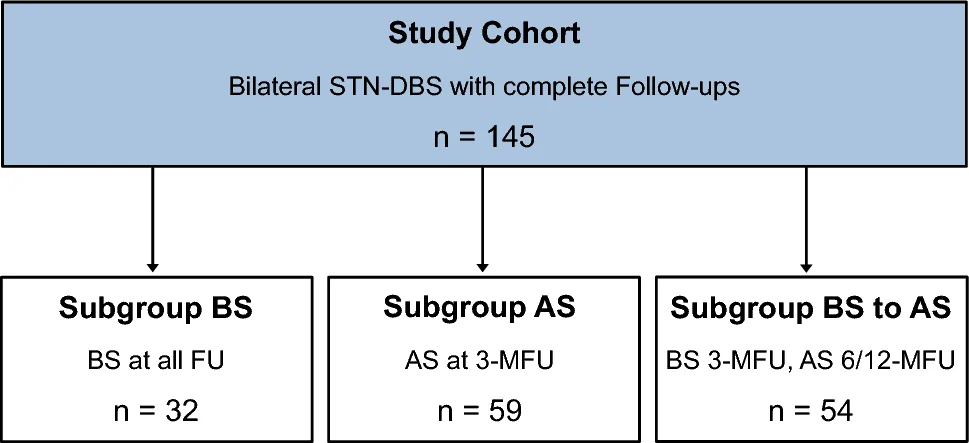
Overview of the study sample. The final study cohort is displayed in blue, while the subgroups are displayed in white. AS = advanced stimulation settings, BS = basic stimulation settings, DBS = deep brain stimulation, FU = follow-up, MFU = months follow-up, STN = subthalamic nucleus

To investigate the effect of a switch to AS on further secondary outcomes, we divided the study cohort in three subgroups (Fig. 1): (i) patients remaining with BS throughout the entire study (“**subgroup BS**”), (ii) patients with advanced settings at the initial postoperative follow-up (“**subgroup AS**”), and (iii) patients with a switch to AS after the 3-MFU (“**subgroup BS to AS**”). For analyzing the LEDD over time within the study cohort as well as the subgroups, we used repeated-measures analysis of variance (ANOVA) and post-hoc paired t-tests or Friedman test for repeated measures followed by pairwise Wilcoxon signed-rank tests if assumptions for parametric testing were not met. Group differences in preoperative levodopa response, CPS at the different follow-up and LEDD reduction were analyzed using a one-way ANOVA with post-hoc independent sample t-test for normally distributed data, or Kruskal-Wallice test with post-hoc Mann–Whitney U test if assumptions for parametric testing were not met. To analyze if certain PD motor subtypes (akinetic-rigid, equivalent or tremor-dominant) were more frequently represented in a certain subgroup, we performed a chi-square test across all subgroups and motor subtypes. Statistical significance was set to p < 0.05. The Shapiro–Wilk test was employed to test for normal data distribution before group comparisons. Mean and standard deviation are reported if not otherwise indicated. Statistical analyses were performed with MATLAB R2025b (The MathWorks Inc., Natick, Massachusetts, United States).

## Results

### Patient characteristics

From 173 screened patients, N = 2 were excluded from the study cohort due to unilateral STN-DBS, N = 6 were excluded because they had revision DBS surgery within the first postoperative year and N = 20 were excluded due to incomplete follow-ups (N = 12 one FU missing, N = 3 two FU missing, N = 5 lost to follow-up). The final study sample consisted of 145 PD patients (study cohort). Patient characteristics of the study cohort are displayed in Table 1. 134 patients had been implanted with Boston Scientific Cartesia™ Directional Leads (n = 47 with Vercise Genus™ R16, n = 2 with Vercise Genus™ P16, n = 74 with Vercise Gevia™, n = 11 with Vercise™ PC), one patient had received Boston Scientific Cartesia™ X Directional Leads (with Vercise Genus™ R32; Boston Scientific, Marlborough, Massachusetts, USA), and 10 patients had been implanted with Medtronic SenSight™ Directional Leads (B33005) and a Percept™ PC neurostimulator (Medtronic, Minneapolis, Minnesota, USA).

**Table 1:** Patient characteristics in the study cohort

**Cohort size **[N]	145 (49 female)
**Age** [years]	63 (± 8.2)
**Disease duration** [years]	9 (IQR 6–12)
**Hoehn and Yahr Stage**	3 (IQR 2.5–3)
**PD motor subtypes** [%, N]	
- akinetic-rigid	49.0 (71)
- equivalent	29.6 (31)
- tremor-dominant	21.4 (43)
**LEDD** [mg]	
- Preoperative	1105.2 (± 438.2)
- 3-MFU	520 (IQR 300–675)
- 6-MFU	492.5 (IQR 285.6–653.1)
- 12-MFU	466 (IQR 250.4–695.6)
**Preoperative UPDRS-III**	
- Medication Off	37.3 (± 11.7)
- Medication On	19.1 (± 8.2)
**Preoperative levodopa response** [%]	46.6 (± 14.5)

IQR = interquartile range, LEDD = levodopa-equivalent daily dose, PD = Parkinson’s disease, UPDRS-III = Unified Parkinson’s Disease Rating Scale-III

### Application of advanced stimulation settings

**Fig. 2:**
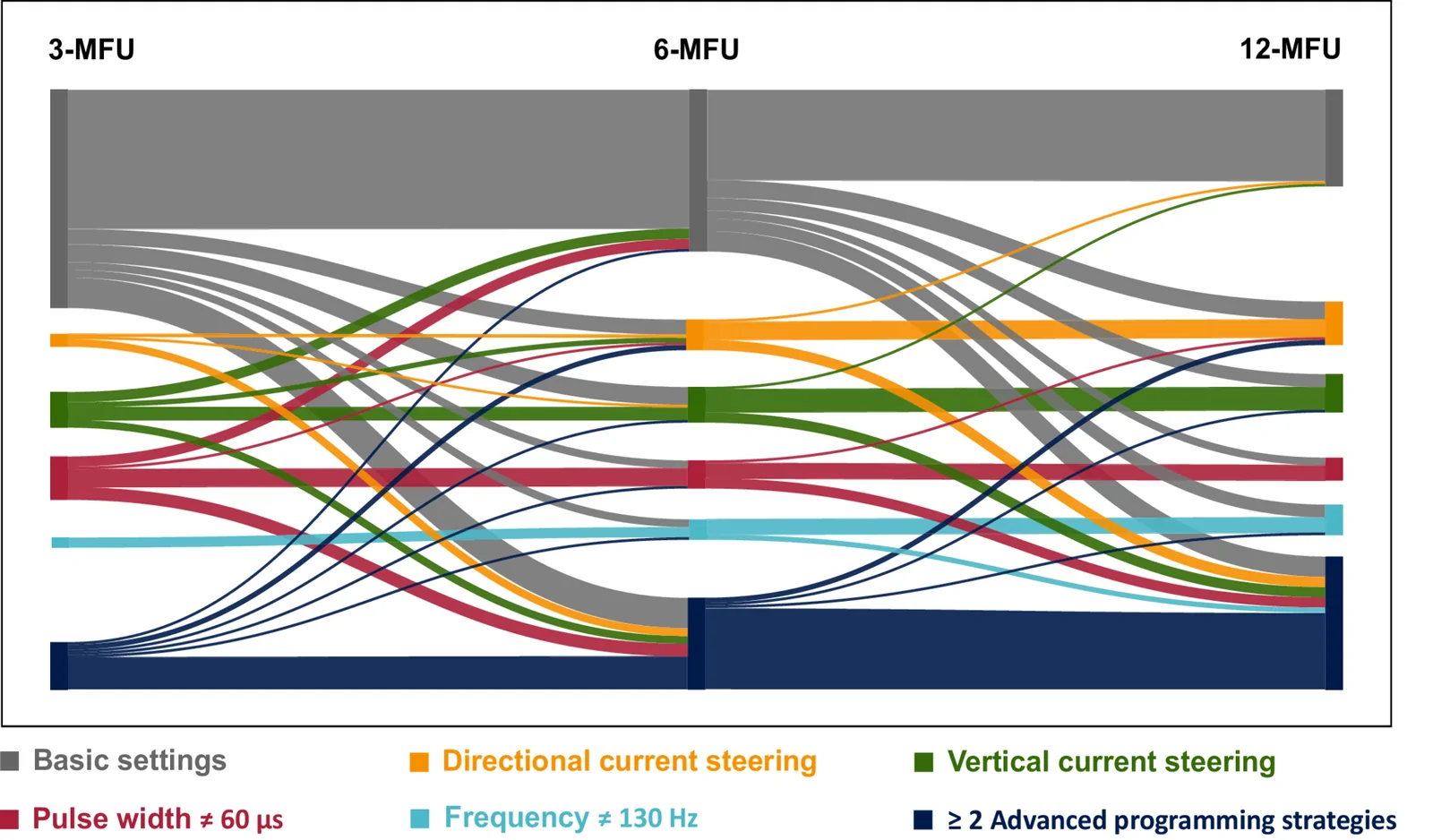
Sankey diagram of the distribution of the different stimulation settings over the three follow-ups (diagram created with SankeyMATIC). MFU = months follow-up

The distribution and changes of the usage of different stimulation settings in the study cohort are illustrated in Table 2 and Fig. 2.

**Table 2:** Percentage distribution of stimulation settings at different follow-ups in the study cohort

**Stimulation settings**	**3-MFU** [%]	**6-MFU** [%]	**12-MFU** [%]	**Estimate** (β)	**OR**	**p-value** (FDR)
**Basic stimulation settings**	59.3 (86)	44.1 (64)	26.2 (38)	**-0.90**	**0.41**	** < 0.001**
**Advanced stimulation settings**	40.7 (59)	55.9 (81)	73.8 (107)	**0.90**	**2.47**	** < 0.001**
**Directional current steering**	10.3 (15)	26.9 (39)	42.1 (61)	**1.08**	**2.95**	** < 0.001**
**Vertical current steering**	18.6 (27)	24.8 (36)	31.7 (46)	**0.43**	**1.54**	**0.006**
**Pulse width ≠ 60 µs** - Pulse width < 60 µs - Pulse width > 60 µs	22.1 (32) 19.3 (28) 2.8 (4)	20.0 (29) 19.3 (28) 0.7 (1)	23.4 (34) 22.1 (32) 1.4 (2)	0.12	1.12	0.495
**Frequency ≠ 130 Hz** - Frequency > 130 Hz - Frequency < 130 Hz	6.9 (10) 6.2 (9) 0.7 (1)	13.8 (20) 12.4 (18) 1.4 (2)	26.8 (39) 19.3 (28) 7.6 (11)	**1.11**	**3.02**	** < 0.001**
**Bipolar**	0.7 (1)	1.4 (2)	1.4 (2)			
**Interleaving**	-	0.7 (1)	1.4 (2)			

**Combination of ≥ 2 AS**	13.1 (19)	24.8 (36)	37.2 (54)	**0.84**	**2.31**	**< 0.001**
- directional + PW	3.4 (5)	4.8 (7)	6.2 (9)			
- directional + FQ	-	1.4 (2)	6.2 (9)			
- directional + vertical	1.4 (2)	7.6 (11)	6.9 (10)			
- directional + bipolar	-	0.7 (1)	-			
- vertical + PW	2.8 (4)	0.7 (1)	2.8 (4)			
- vertical + FQ	1.4 (2)	2.1 (3)	1.4 (2)			
- frequency + PW	0.7 (1)	1.4 (2)	0.7 (1)			
- vertical + PW + FQ	1.4 (2)	1.4 (2)	1.4 (2)			
- vertical + directional + PW	0.7 (1)	2.8 (4)	3.4 (5)			
- vertical + directional + FQ	0.7 (1)	-	2.8 (4)			
- vertical + directional + bipolar	0.7 (1)	-	-			
- directional + FQ + bipolar	-	0.7 (1)	1.4 (2)			
- directional + PW + FQ	-	0.7 (1)	0.7 (1)			
- directional + FQ + interleaving	-	-	0.7 (1)			
- directional + vertical + PW + FQ	-	-	2.1 (3)			
- vertical + interleaving + PW + FQ	-	0.7 (1)	0.7 (1)			

The absolute number is displayed in brackets. Results of the mixed-effects logistic regressions for changes of the application of each different stimulation settings over time with corresponding estimate, odds ratio and p-value. AS = advanced settings, FDR = false discovery rate, FQ = frequency, MFU = months follow-up, OR = odds ratio, PW = pulse width. Significant (p < 0.05) changes over time are marked in bold

Mixed-effects logistic regressions revealed a significant decrease in basic stimulation settings over time (OR 0.41, p < 0.001), while overall advanced stimulation settings increased significantly (OR 2.47, p < 0.001). Directional and vertical stimulation as well as variations in frequency (including frequencies > 130 Hz and < 130 Hz) significantly increased over time (Table 2).

In most patients (77.9%, N = 113), AS were applied at least once during the first postoperative year. A change of strategy was performed in 41.4% between the 3- and 6-MFU and in 33.8% between the 6- and 12-MFU. At 12-MFU, 26.2% of patients remained in BS while 73.8% were programmed using AS and 37.2% used at least two different AS simultaneously.

The most frequently used AS was directional current steering followed by vertical current steering. If two or more levels were activated, they were mostly adjacent levels, and in only very few cases (N = 3) two non-adjacent levels. At 6-MFU, variation in pulse width was more common than frequency variation, while this finding reversed at 12-MFU. Bipolar stimulation was used only in three patients and interleaving/independent frequency stimulation in two patients.

Most common AS combinations at 12-MFU were directional DBS with variations in pulse width (6.2%, N = 9), frequency (6.2%, N = 9) or directional DBS in combination with vertical current steering (6.9%, N = 10). Our cohort’s pulse widths varied from 30 – 90 µs, while frequencies ranged from 60 – 204 Hz. The current distribution over the lead at the follow-ups is illustrated in Additional file 1.

### LEDD reduction and lead positions

Mean LEDD was significantly reduced in the study cohort at all three follow-ups compared to baseline with no further significant change between the follow-up periods (Table 1, all compared to pre-DBS p < 0.001).

**Fig. 3:**
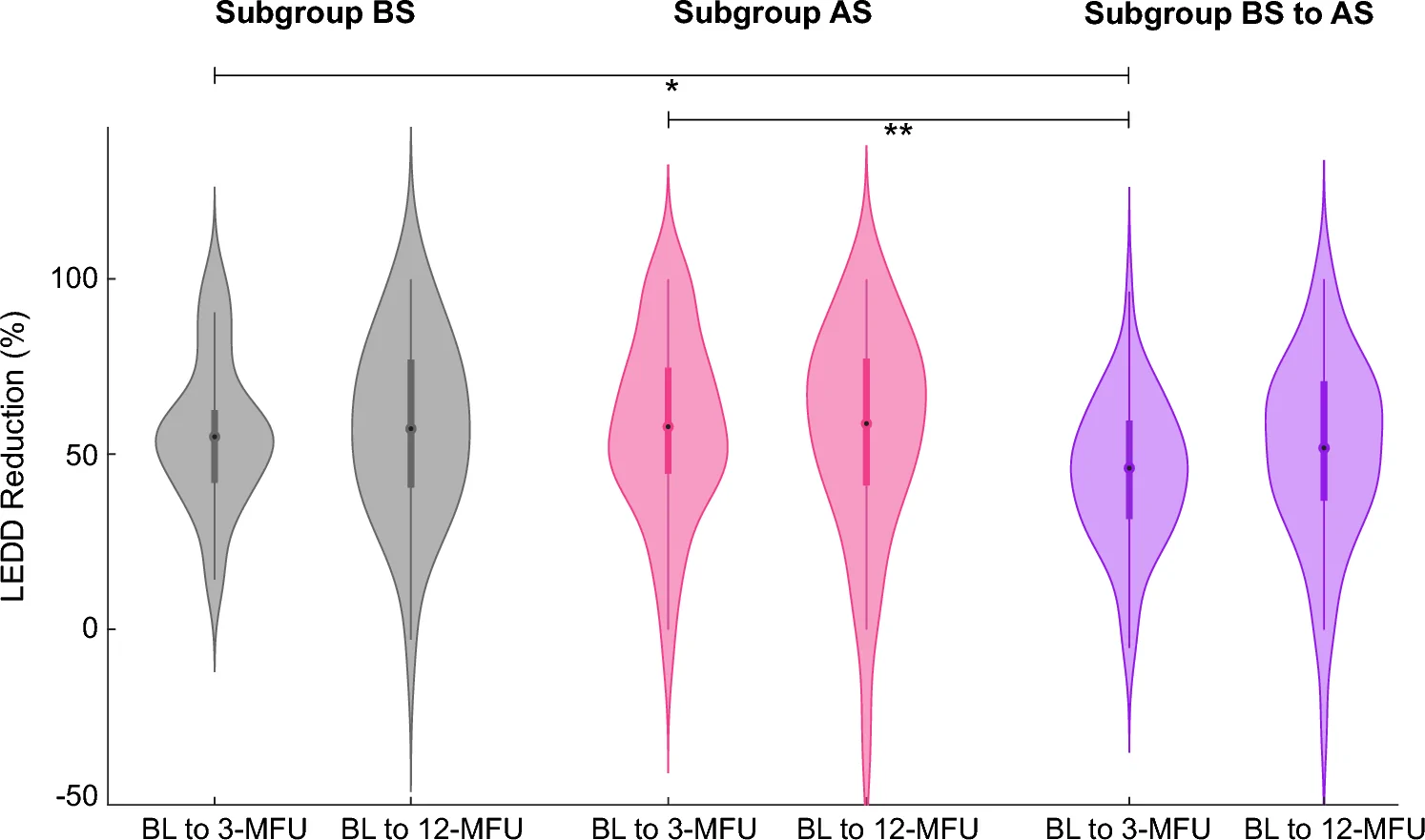
Change scores of levodopa-equivalent daily dose (LEDD) within the different subgroups (grey: subgroup BS, pink: subgroup AS, purple: subgroup BS to AS) from baseline to 3-MFU and from baseline to 12-MFU. AS = advanced stimulation settings, BL = baseline, BS = basic stimulation settings, MFU = months follow-up. * p = 0.013, ** p = 0.003

The median distance to the motor STN center of gravity was larger for leads with AS at least once (N = 212, 1.9 mm (IQR 1.3—2.5 mm)) than for leads with BS at all follow-ups (N = 76, 1.5 mm (IQR 1.1—2.0 mm), p = 0.002). 95% confidence ellipsoids of the central coordinates of the two groups are displayed in Fig. 3. For comparability, we excluded the patient with Boston Scientific Cartesia™ X Directional Leads for the analysis of central contact coordinates.

### Subgroup analysis

Similar to the study cohort, LEDD was significantly reduced from baseline to all follow-ups without significant differences between the follow-ups in all subgroups (Table 3, all compared to pre-DBS p < 0.001). Demographic data as well as clinical outcome data of patients of the three different subgroups are displayed in Table 3.

**Table 3:** Patient characteristics and clinical outcome data within the subgroups

Patient characteristics	Subgroup BS	Subgroup AS	Subgroup BS to AS
**Cohort size** [N]	32	59	54
**Age** [years]	64.3 (± 8.3)	61.8 (± 9.1)	63.6 (± 6.9)
**Disease duration** [years]	8 (6–14)	9.6 (± 4.1)	9 (6–11)
**Hoehn and Yahr Stage**	3 (2.5–3.5)	3 (2–3.75)	3 (2.5–3)
**PD motor subtypes** [%, N]			
- akinetic-rigid	68.75 (22)	45.8 (27)	40.7 (22)
- equivalent	18.75 (6)	30.5 (18)	35.2 (19)
- tremor-dominant	12.5 (4)	23.7 (14)	24.1 (13)
**Preoperative UPDRS-III**			
- Medication Off	33.9 (± 11.1)	38.6 (± 12.3)	37.8 (± 11.2)
- Medication On	16.2 (± 8.2)	20.5 (± 9.1)	19.4 (± 6.8)
**Preoperative levodopa response** [%]	54.6 (± 16.1)	47.9 (± 15.0)	48.4 (± 12.4)
**LEDD** [mg]			
- Pre-DBS	1192.2 (± 370.5)	1033.3 (± 472.2)	1132.1 (± 431.7)
- 3-MFU	579 (362.7–704.1)	416.5 (190.9–624.5)	564.7 (450.9–675)
- 6-MFU	472.4 (± 238.8)	413.2 (± 275.6)	527.5 (340–713.4)
- 12-MFU	488.1 (± 264.4)	400.5 (160.5–696.9)	487.7 (340–742.5)
**LEDD Change** [%]			
- Pre-DBS to 3-MFU	55.8 (± 20.9)	57.5 (± 25.6)	44.3 (± 19.9)
- Pre-DBS to 6-MFU	59.9 (± 20.8)	58.9 (± 25.6)	48.3 (± 22.8)
- Pre-DBS to 12-MFU	58 (± 24.3)	56.4 (± 30.2)	52.2 (± 25.8)
**CPS** [µC/s]			
- 3-MFU	10.7 (8.4–14.2)	14.3 (11.3–17.2)	12.3 (10.1–15.6
- 6-MFU	12.5 (11.1–16.2)	16.1 (12.3–19.5)	15.0 (11.7–17.2)
- 12-MFU	14.4 (12.1–17.8)	18.7 (13.4–21.8)	16.3 (12.0–21.5)

Mean and standard deviation (±) or median with interquartile range are displayed. AS = advanced stimulation settings, BS = basic stimulation settings, CPS = Charge per second, DBS = deep brain stimulation, LEDD = levodopa-equivalent daily dose, MFU = months follow-up, PD = Parkinson’s disease, UPDRS-III = Unified Parkinson’s Disease Rating Scale-III

Chi-square test showed no significant difference in distribution of the three subgroups between PD motor subtypes (p = 0.147), although the majority of patients treated with basic settings at all follow-ups had akinetic-rigid PD (68.75%).

There was no difference in preoperative levodopa response between the three groups (p = 0.08), although patients in the “subgroup AS” and “subgroup BS to AS” had a tendency to slightly lower levodopa improvements (“subgroup AS” 47.9% (± 15.0), “subgroup BS to AS” 48.4% (± 12.4), “subgroup BS” 54.6% (± 16.1)).

**Fig. 4:**
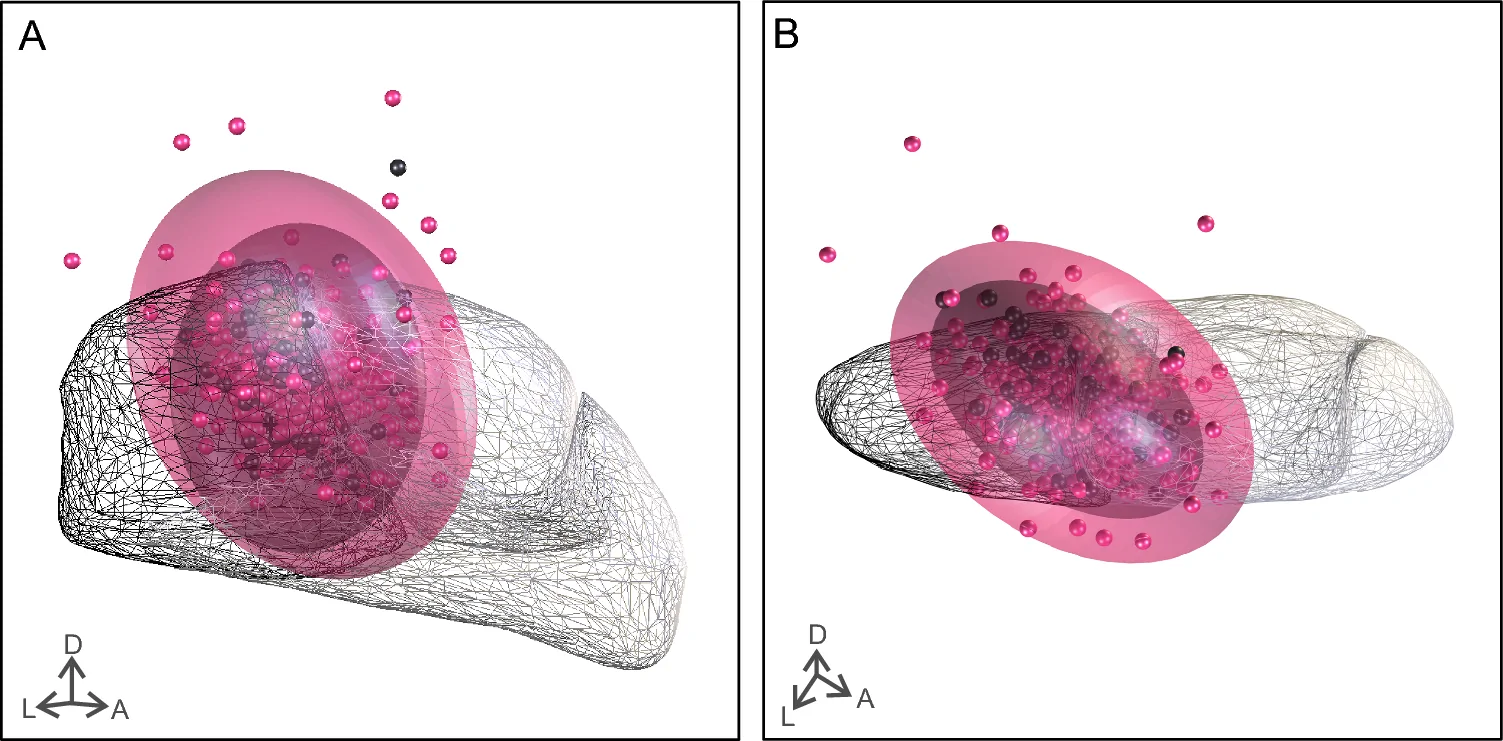
Mean contact coordinates (small balls) and 95% confidence ellipsoids in leads with basic settings (grey) vs. leads with advanced stimulation settings (pink). The motor STN is displayed as a black mash, associative and limbic STN are displayed as a gray mesh. **A** antero-lateral view along the STN axis, **B** dorso-lateral view of the STN. A = anterior, D = dorsal, L = lateral

As displayed in Fig. 4, patients treated with AS after the 3-MFU had significantly lower initial LEDD reduction (“subgroup BS to AS”) than patients who remained with basic settings (“subgroup BS”, p = 0.013) or patients who were initially treated with AS (“subgroup AS”, p = 0.003). Similar results were seen at the 6-MFU: patients with a change to AS after the 3-MFU had significantly smaller initial LEDD reduction (“subgroup BS to AS”) than patients with BS at all follow-ups (“subgroup BS”, p = 0.021) or patients initially programmed with AS (“subgroup AS”, p = 0.022). However, no significant differences were observed at the 12-MFU between all groups (p = 0.283).

Regarding the applied CPS, a lower median CPS was found at the 3-MFU for patients in the “subgroup BS” as compared to patients initially treated with AS (“subgroup AS”, p = 0.001). No differences were found between these groups and the “subgroup BS to AS” (p = 0.156 and p = 0.054).

No differences in the median CPS were found at the other follow-ups between all subgroups (6-MFU: p = 0.066, 12-MFU: p = 0.108).

## Discussion

This retrospective longitudinal study demonstrates that AS are frequently used in clinical routine in a specialized DBS center for the treatment of PD patients with STN-DBS. Directional and vertical current steering were the most frequently applied AS in our cohort, but also combinations of different AS were common. Over time, basic stimulation settings are used less frequently, while the application of advanced settings as well as combinations of AS increase.

When observing the current distribution along the lead in the study cohort, both middle levels were activated in 88.9% during the long-term follow-up. This might be of interest since the application of novel DBS devices capable of so called “adaptive stimulation” is to date only possible with specific combinations of contacts used for stimulation or sensing respectively.

Bipolar and interleaving/independent frequency stimulation were only applied in combination with other AS, indicating that those settings might only have been considered if other AS were insufficient.

Although baseline characteristics and distribution of motor subtypes between the subgroups did not differ, patients switched to AS after 3 months had a lower initial LEDD reduction than patients switched earlier to AS or staying in BS throughout the follow-up period. However, after switching to AS, the LEDD reduction achieved at the 12-MFU aligned between groups, without a significant difference in CPS as a marker of the applied stimulation energy.

Interestingly, leads at whom AS were applied had a slightly larger distance to the motor STN than leads at whom only BS were applied. This leads to the careful conclusion that AS might have the potential to optimize the effect of STN-DBS in patients with PD. Of note, differences in lead placement were only marginal and do not reflect a general misplacement of electrodes in patients in whom AS were applied.

Some limitations apply to this study. This study only represents experiences from one DBS center in Germany and might not represent the application of different stimulation settings in other centers. Despite guidelines, programming practices vary widely between centers. Another major limitation of the study is its retrospective nature without systematic documentation of reasons for adaptation of stimulation settings. Therefore, our observations cannot be used to establish causality for changing stimulation strategies. Additionally, there were no motor assessments in medication off state recorded for each follow-up, not allowing to investigate the effect of changes in stimulation settings on specific motor symptoms.

To guide the reader to the best of our knowledge as implemented in our clinical routine, the following section provides an overview of current literature on advanced stimulation settings and an overall summary of prospective trials regarding the different advanced stimulation settings (Additional file 2).

### Overview of advanced stimulation settings

***Directional current steering***, the most frequently applied AS in our cohort, is known to increase the therapeutic window mainly by increasing side effect thresholds, which is especially helpful in slightly inaccurate lead placement [2–4]. A recent meta-analysis confirmed the findings on side effect improvement but did not find motor improvement under directional DBS compared to omnidirectional settings [34]. In a study by Schnitzler et al., directional stimulation was preferred by most patients over omnidirectional stimulation [5]. This was also reported regarding the long-term follow-up [35, 36]. Further advantage of directional current steering can be reduced required electrical current, which could increase battery longevity [5]. In a survey-based evaluation of reasons for changing from omnidirectional to directional stimulation, the main two reasons were “burdensome side effects” and “clinically suboptimal effect” [37]. Previously published experiences from other centers showed that directional steering is applied frequently in the long-term follow-up [35, 38]. Karl and colleagues found similar usages of directional DBS 12-months postoperatively (39%; in our cohort: 42.1%) [38]. Future randomized double-blinded studies are needed to verify the advantages of directional current steering [39].

With ***vertical current steering*** it is possible to distribute the current longitudinally over the lead to allow a more specific and individual stimulation setting by shaping the current more precisely with different amplitudes at different levels to fit the target area [40]. This enables improvement of symptoms that could not be treated by activating a single contact due to limiting side effects induced by current spread beyond the target area [6, 41]. Vertical current steering can reduce the total electrical energy delivered while maintaining therapeutic efficacy [42]. In our cohort, it was the second most commonly used AS, with 31.7% at long-term follow-up.

Continuous deep brain stimulation is usually applied as high-frequency stimulation at 130 Hz [1]. Some studies have focused on the variation of ***stimulation frequencies***. The evidence on low frequency stimulation (60 – 85 Hz) suggests a positive effect on axial symptoms such as gait and postural stability [14, 16, 43]. Further evidence points to an improvement in swallowing and speech with low-frequency stimulation [43, 44]. Very low frequencies (theta) might positively impact verbal fluency and cognition [45, 46]. In contrast, tremor worsens with low frequencies and might respond better to frequencies > 130 Hz [14, 43, 47]. In our cohort, frequency variation was chosen in 26.8% in the long-term follow-up, mostly with frequencies > 130 Hz (71.8%).

***Pulse width*** has historically been set to 60 µs, which was the lowest possible setting in early DBS devices [1, 19]. Since introducing devices allowing for lower pulse widths, studies have shown that decreasing the pulse width can widen the therapeutic window, supposedly due to a greater increase in the side effect threshold compared to the effect threshold, [10, 12, 48] while efficacy ought to be noninferior to 60 µs [9, 13]. This benefit has also been shown for the long-term [36]. A lower pulse width might also positively influence speech and gait parameters [10, 11]. It has further been proposed that lower pulse widths are more energy efficient, although this has been controversially discussed [9, 10, 12]. In our cohort, pulse widths were changed in 23.4% of patients in the long-term follow-up, mostly with decreased pulse widths (94.1%).

By default, current is applied with a monopolar cathodic stimulation while the anode is placed on the implantable pulse generator [19]. ***Bipolar stimulation*** with both cathode and anode placed on the lead is a possible alternative. Compared to cathodic monopolar stimulation, bipolar stimulation has a smaller therapeutic effect, requiring a higher stimulation intensity [19]. However, bipolar stimulation may result in a wider therapeutic window with a higher side-effect threshold [18]. It can be a good alternative in case of limiting side effects. In our cohort, bipolar stimulation was only used in three patients.

During ***interleaving stimulation,*** two different stimulation programs are applied on the same lead using either alternating pulses at the same frequency or independently at different frequencies (= independent frequency stimulation). Interleaving stimulation might be advantageous if limiting side effects occur [41]. It might stimulate different beneficial neuroanatomical pathways or regions, such as pallidofugal fibers or the zona incerta, positively impacting burdensome dyskinesias while also applying current to the motor STN [49, 50]. Interleaving stimulation might thereby also increase efficacy [49]. By combining two low-frequency programs with a partially overlapping high-frequency area (interleave-interlink), axial symptoms could improve without worsening appendicular symptoms [20, 22]. Interleaving stimulation was only applied in two patients in our cohort during the first postoperative year.

Both bipolar and interleaving stimulation were only applied in combination with other AS. This might indicate that those strategies were only considered if other AS were insufficient.

## Conclusion

Advanced stimulation settings are frequently used in clinical routine and might be advantageous for symptom control, possibly compensating for variations in lead positions. Future prospective studies are warranted to establish clear paradigms and indications for applying advanced stimulation settings, also considering adaptive DBS, in chronic DBS to optimize individual benefit, helping clinicians anticipate which patients might benefit after switching stimulation settings. Updated algorithms are required to help clinicians decide on when to consider which strategy.

## Supplementary Information

Additional file 1.

Additional file 2.

## Data Availability

The datasets used and analyzed during the current study are available from the corresponding author on reasonable request.

## Abbreviations

ANOVA: Analysis of variance
AS: Advanced stimulation settings
BS: Basic stimulation settings
CPS: Charge per second
DBS: Deep brain stimulation
DISTAL: DBS intrinsic template
FDR: False discovery rate
IQR: Interquartile range
LEDD: Levodopa-equivalent daily dose
MFU: Months follow-up
OR: Odds ratio
PD: Parkinson’s disease
STN: Subthalamic nucleus
UPDRS-III: Unified parkinson’s disease rating scale-III
