# Publisher Correction: Stimulation settings in subthalamic nucleus deep brain stimulation for parkinson’s disease – a retrospective single-center observational study

**DOI:** 10.1186/s42466-026-00517-0

**Published:** 2026-08-25

**Authors:** Charlotte Schedlich-Teufer, Gregor A. Brandt, Christina van der Linden, Hannah Jergas, Juan Carlos Baldermann, Vasilija Stopic, Gereon R. Fink, Veerle Visser-Vandewalle, Till A. Dembek, Michael T. Barbe, Jan Niklas Petry-Schmelzer

**Affiliations:** 1 Department of Neurology, Faculty of Medicine and University Hospital Cologne, University of Cologne, Cologne, Germany; 2 Department of Neurology With Experimental Neurology, Charité - Universitätsmedizin Berlin, corporate member of Freie Universität Berlin, Humboldt Universität Zu Berlin, Berlin, Germany; 3 Department of Psychiatry and Psychotherapy, Medical Center - University of Freiburg, Faculty of Medicine, University of Freiburg, Freiburg, Germany; 4 Cognitive Neuroscience, Institute for Neuroscience and Medicine (INM-3), Jülich Research Center, Jülich, Germany; 5 Department of Stereotactic and Functional Neurosurgery, Faculty of Medicine and University Hospital Cologne, University of Cologne, Cologne, Germany

**Publisher Correction: Neurological Research and Practice (2026) 8:53**

10.1186/s42466-026-00477-5

During the incorporation of the proof corrections, some of the rows in Tables 1 and 3 seem to have been inadvertently shifted.

Additionally, Table 3; Fig. 3 are now displayed across the entire width of the page for better readability.

Table 1 in the original article:

**Table 1:** Patient characteristics in the study cohort

**Cohort size** [N]	145 (49 female)
**Age** [years]	63 (± 8.2)
**Disease duration** [years]	9 (IQR 6–12)
**Hoehn and Yahr Stage**	3 (IQR 2.5–3)
**PD motor subtypes** [%, N] –akinetic-rigid –equivalent –tremor-dominant	49.0 (71) 29.6 (31) 21.4 (43)
**LEDD** [mg] - Preoperative - 3-MFU - 6-MFU - 12-MFU	1105.2 (± 438.2) 520 (IQR 300–675) 492.5 (IQR 285.6–653.1) 466 (IQR 250.4–695.6)
**Preoperative UPDRS-III** - Medication Off - Medication On	37.3 (± 11.7) 19.1 (± 8.2)
**Preoperative levodopa response** [%]	46.6 (± 14.5)

IQR = interquartile range, LEDD = levodopa-equivalent daily dose, PD = Parkinson’s disease, UPDRS-III = Unified Parkinson’s Disease Rating Scale-III

Corrected Table 1:

**Table 1:** Patient characteristics in the study cohort

**Cohort size** [N]	145 (49 female)
**Age** [years]	63 (± 8.2)
**Disease duration** [years]	9 (IQR 6–12)
**Hoehn and Yahr Stage**	3 (IQR 2.5–3)
**PD motor subtypes** [%, N]	
- akinetic-rigid - equivalent - tremor-dominant	49.0 (71) 29.6 (31) 21.4 (43)
**LEDD** [mg]	
- Preoperative - 3-MFU - 6-MFU - 12-MFU	1105.2 (± 438.2) 520 (IQR 300–675) 492.5 (IQR 285.6–653.1) 466 (IQR 250.4–695.6)
**Preoperative UPDRS-III**	
- Medication Off - Medication On	37.3 (± 11.7) 19.1 (± 8.2)
**Preoperative levodopa response** [%]	46.6 (± 14.5)

IQR = interquartile range, LEDD = levodopa-equivalent daily dose, PD = Parkinson’s disease, UPDRS-III = Unified Parkinson’s Disease Rating Scale-III

Table 3 in the original article:

**Table 3:** Patient characteristics and clinical outcome data within the subgroups

Patient characteristics	Subgroup BS	Subgroup AS	Subgroup BS to AS
**Cohort size** [N]	32	59	54
**Age** [years]	64.3 (± 8.3)	61.8 (± 9.1)	63.6 (± 6.9)
**Disease duration** [years]	8 (6–14)	9.6 (± 4.1)	9 (6–11)
**Hoehn and Yahr Stage**	3 (2.5–3.5)	3 (2–3.75)	3 (2.5–3)
**PD motor subtypes** [%, N] –akinetic-rigid –equivalent –tremor-dominant	68.75 (22) 18.75 (6) 12.5 (4)	45.8 (27) 30.5 (18) 23.7 (14)	40.7 (22) 35.2 (19) 24.1 (13)
**Preoperative UPDRS-III** - Medication Off - Medication On	33.9 (± 11.1) 16.2 (± 8.2)	38.6 (± 12.3) 20.5 (± 9.1)	37.8 (± 11.2) 19.4 (± 6.8)
**Preoperative levodopa response** [%]	54.6 (± 16.1)	47.9 (± 15.0)	48.4 (± 12.4)
**LEDD** [mg] - Pre-DBS - 3-MFU - 6-MFU - 12-MFU	1192.2 (± 370.5) 579 (362.7–704.1) 472.4 (± 238.8) 488.1 (± 264.4)	1033.3 (± 472.2) 416.5 (190.9–624.5) 413.2 (± 275.6) 400.5 (160.5–696.9)	1132.1 (± 431.7) 564.7 (450.9–675) 527.5 (340–713.4) 487.7 (340–742.5)
**LEDD Change** [%] - Pre-DBS to 3-MFU - Pre-DBS to 6-MFU - Pre-DBS to 12-MFU	55.8 (± 20.9) 59.9 (± 20.8) 58 (± 24.3)	57.5 (± 25.6) 58.9 (± 25.6) 56.4 (± 30.2)	44.3 (± 19.9) 48.3 (± 22.8) 52.2 (± 25.8)
**CPS** [µC/s] - 3-MFU - 6-MFU - 12-MFU	10.7 (8.4–14.2) 12.5 (11.1–16.2) 14.4 (12.1–17.8)	14.3 (11.3–17.2) 16.1 (12.3–19.5) 18.7 (13.4–21.8)	12.3 (10.1–15.6 15.0 (11.7–17.2) 16.3 (12.0–21.5)

Mean and standard deviation (±) or median with interquartile range are displayed. AS = advanced stimulation settings, BS = basic stimulation settings, CPS = Charge per second, DBS = deep brain stimulation, LEDD = levodopa-equivalent daily dose, MFU = months follow-up, PD = Parkinson’s disease, UPDRS-III = Unified Parkinson’s Disease Rating Scale-III

Corrected Table 3:

**Table 3:** Patient characteristics and clinical outcome data within the subgroups

Patient characteristics	Subgroup BS	Subgroup AS	Subgroup BS to AS
**Cohort size** [N]	32	59	54
**Age** [years]	64.3 (± 8.3)	61.8 (± 9.1)	63.6 (± 6.9)
**Disease duration** [years]	8 (6–14)	9.6 (± 4.1)	9 (6–11)
**Hoehn and Yahr Stage**	3 (2.5–3.5)	3 (2–3.75)	3 (2.5–3)
**PD motor subtypes** [%, N]			
- akinetic-rigid - equivalent - tremor-dominant	68.75 (22) 18.75 (6) 12.5 (4)	45.8 (27) 30.5 (18) 23.7 (14)	40.7 (22) 35.2 (19) 24.1 (13)
**Preoperative UPDRS-III**			
- Medication Off - Medication On	33.9 (± 11.1) 16.2 (± 8.2)	38.6 (± 12.3) 20.5 (± 9.1)	37.8 (± 11.2) 19.4 (± 6.8)
**Preoperative levodopa response** [%]	54.6 (± 16.1)	47.9 (± 15.0)	48.4 (± 12.4)
**LEDD **[mg]			
- Pre-DBS - 3-MFU - 6-MFU - 12-MFU	1192.2 (± 370.5) 579 (362.7–704.1) 472.4 (± 238.8) 488.1 (± 264.4)	1033.3 (± 472.2) 416.5 (190.9–624.5) 413.2 (± 275.6) 400.5 (160.5–696.9)	1132.1 (± 431.7) 564.7 (450.9–675) 527.5 (340–713.4) 487.7 (340–742.5)
**LEDD Change** [%]			
- Pre-DBS to 3-MFU - Pre-DBS to 6-MFU - Pre-DBS to 12-MFU	55.8 (± 20.9) 59.9 (± 20.8) 58 (± 24.3)	57.5 (± 25.6) 58.9 (± 25.6) 56.4 (± 30.2)	44.3 (± 19.9) 48.3 (± 22.8) 52.2 (± 25.8)
**CPS** [µC/s]			
- 3-MFU - 6-MFU - 12-MFU	10.7 (8.4–14.2) 12.5 (11.1–16.2) 14.4 (12.1–17.8)	14.3 (11.3–17.2) 16.1 (12.3–19.5) 18.7 (13.4–21.8)	12.3 (10.1–15.6 15.0 (11.7–17.2) 16.3 (12.0–21.5)

Mean and standard deviation (±) or median with interquartile range are displayed. AS = advanced stimulation settings, BS = basic stimulation settings, CPS = Charge per second, DBS = deep brain stimulation, LEDD = levodopa-equivalent daily dose, MFU = months follow-up, PD = Parkinson’s disease, UPDRS-III = Unified Parkinson’s Disease Rating Scale-III

The original article has been corrected.

