# Supplementary material for: Stimulation settings in subthalamic nucleus deep brain stimulation for parkinson’s disease – a retrospective single-center observational study
Source: Neurol Res Pract. 2026 Jul 2;8(1):53. doi: 10.1186/s42466-026-00477-5 (PMC13330291; doi:10.1186/s42466-026-00477-5)
Supplement: Supplementary file 1 — Additional file 1. [file 42466_2026_477_MOESM1_ESM.pdf]

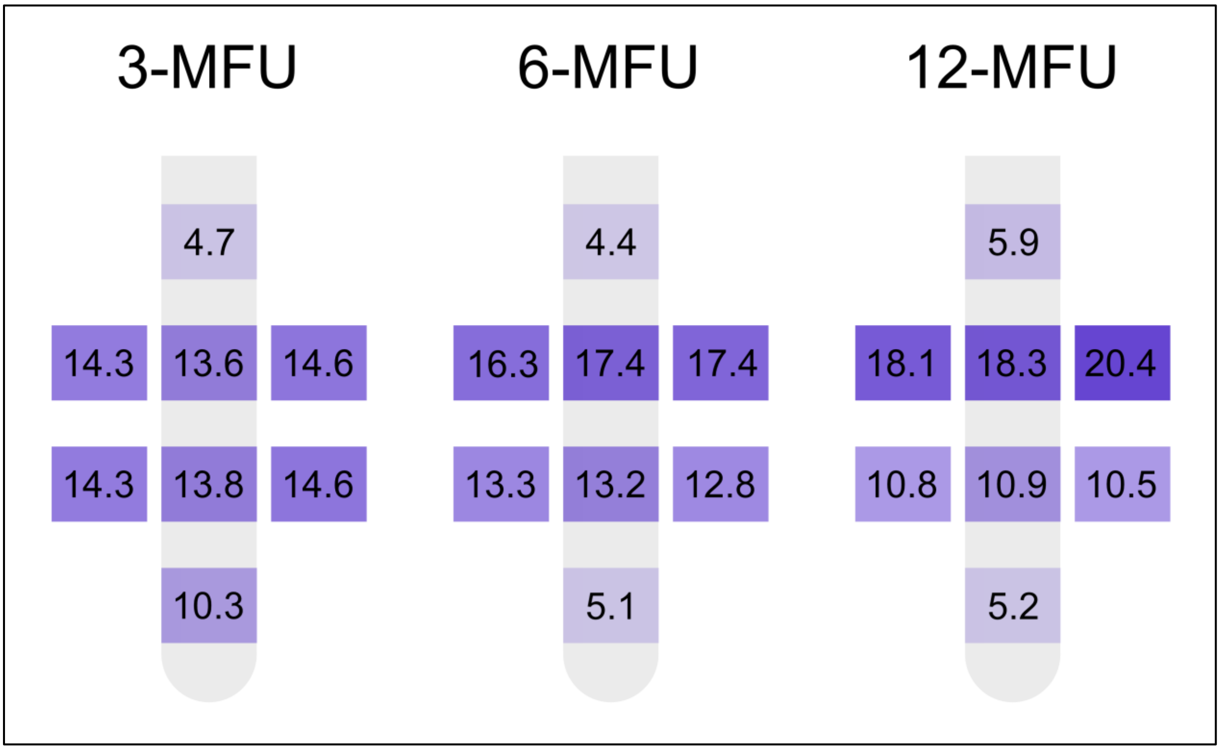


**Additional file 1.** Percentage distribution of activated contacts of the lead at all follow-ups. MFU = months follow-up.
