# Supplementary material for: Stimulation settings in subthalamic nucleus deep brain stimulation for parkinson’s disease – a retrospective single-center observational study
Source: Neurol Res Pract. 2026 Jul 2;8(1):53. doi: 10.1186/s42466-026-00477-5 (PMC13330291; doi:10.1186/s42466-026-00477-5)
Supplement: Supplementary file 2 — Additional file 2. [file 42466_2026_477_MOESM2_ESM.pdf]

Additional file 2 Overview of prospective studies focusing on advanced stimulation settings

Author (Year)	Journal	Study design	No. of patients	Outcome
**Directional current steering**				
Contarino et al. (2014)[1]	Neurology	Randomized, double-blind	8	Increased TW and higher SE thresholds
Dayal et al. (2020)[2]	Mov Disord.	Randomized, double-blind	32	Lower efficacy threshold, higher SE threshold and larger TW, higher Speech Intelligibility, lower dyskinesias
Dembek et al. (2017)[3]	Mov Disord.	Randomized, double-blind	10	Increased TW, higher SE thresholds, equivalent motor efficacy
Hurt et al. (2025)[4]	J Parkinsons Dis.	Randomized, double-blind	31	Better motor performance compared to ring-mode stimulation in unilateral DBS, increased TW, higher SE thresholds
Pollo et al. (2014)[5]	Brain	Randomized, double-blind	11	Increased TW, lower effect threshold
Schnitzler et al. (2022)[6]	Neuromodulation	Randomized, double-blind	234	Increased TW, higher SE thresholds, reduced therapeutic current strength; directional DBS preferred over omnidirectional DBS by patients and clinicians
Sekimoto et al. (2023)[7]	Parkinsonism Relat Disord.	Randomized, double-blind	11	Stride length worsens while steering towards the internal capsule
Steigerwald et al. (2016)[8]	Mov Disord.	Non-randomized, non-blinded	7	Increased TW
**Multi-level contact configuration**				
Hui et al. (2020)[9]	Sci Rep.	Randomized, non-blinded	7	Reduced TEED with multi-level contact configuration

**Variation of frequency**				
Annic et al. (2014)[10]	J Parkinsons Dis.	Randomized, double-blind	22	Patients with higher age, severe axial phenotype five years after surgery, lower levodopa responsiveness on akinesia preoperatively and on the axial subscore one year postoperatively might respond to LFS
Busteed et al. (2024)[11]	Arch Clin Neuropsychol.	Controlled, non-randomized, single-blind	38	HFS negatively affected letter, semantic and action fluency; LFS did not affect verbal fluency
Cheng et al. (2024)[12]	Acta Neurochirurgica	Non-randomzied, non-blinded	21	HFS (130 - 185 Hz) did not improve axial symptoms; LFS (60 - 90 Hz) and VFS stimulation improved axial symptoms; lower tremor control during LFS
Conway et al. (2021)[13]	J NeuroEngineering Rehabil.	Randomized, double-blind	14	LFS (60 Hz) while maintaining TEED improved medial-lateral and vertical trunk rhythmicity compared to HFS; tremor might worsen under LFS
Dutke et al. (2025)[14]	J Parkinsons Dis.	Controlled, randomized, double-blind	15	Benefits in subjective perception of gait in a subgroup of patients with theta burst DBS (interburst frequency 5 Hz, intraburst frequency 200 Hz) compared to conventional DBS with slightly different movement-induced alpha and beta suppression patterns in premotor/motor cortex
Fagundes et al. (2016)[15]	Parkinsons Dis.	Randomized, double-blind	20	Better phonemic and action verbal fluency during LFS (60 Hz) compared to HFS (130 Hz)
Grover et al. (2019)[16]	J Parkinsons Dis.	Randomized, double-blind	15	Speech intelligibility (articulation, respiration, phonation, and prosody) and phonemic verbal fluency switching improved with LFS compared to HFS
Herz et al. (2025)[17]	Mov Disord.	Randomized, single-blind	24	low frequency deep brain stimulation (4 Hz) during early NREM sleep improved overnight memory retention

Imbalzano et al. (2025)[18]	Mov Disord.	Randomized, double-blind	12	Non-episodic and episodic verbal fluency improved with theta-gamma stimulation (100-180 Hz + 6 Hz); no effect on phonemic and switching fluency; no motor worsening, only mild adverse events
Jia et al. (2024)[19]	Nat Sci Rev.	Non-randomized, double-blind	28	VFS improved gait speed, reduced freezing episodes, and improved tremor, bradykinesia, and rigidity with a sustained effect after 6 and 12 months
Khoo et al. (2014)[20]	Mov Disord.	Randomized, double-blind	14	LFS (60 Hz) was superior in improving the UPDRS motor score and axial and akinesia subscores compared to 130 Hz; optimal LFS contacts were more ventrally located
Lee et al. (2021)[21]	Brain Stimulation	Randomized, single-blind	9	Acute left dorsal peak theta frequency STN stimulation improved episodic category verbal fluency
Moreau et al. (2008)[22]	Neurology	Randomized, double-blind	13	LFS (60 Hz) while maintaining TEED reduced FOG episodes compared to 130 Hz
Moreau et al. (2011)[23]	Mov Disord.	Randomized, double-blind	11	Aerodynamic speech parameters and speech intelligibility improved during LFS (60 Hz) compared to HFS (130 Hz)
Moro et al. (2002)[24]	Neurology	Randomized, double-blind	12	Frequencies ≥ 50 Hz improved bradykinesia and tremor with maximum benefit at 185 Hz; no significant difference between 130 and 185 Hz
Mügge et al. (2023)[25]	Neurol Res Pract.	Randomized	23	85 Hz improved gait speed, stride length, and leg lift compared to 130 and 30 Hz
Phibbs et al. (2014)[26]	Neuromodulation	Randomized, double-blind	20	No difference in stride length between LFS (60 Hz) and HFS (130 Hz) with lower tremor control during LFS
Ricchi et al. (2012)[27]	Brain Stimulation	Non-randomized, partly blind	11	LFS (80 Hz) while maintaining TEED had an immediate positive effect on gait with no improvement in the long-term follow-up
Ricciardi et al. (2025)[28]	Mov Disord.	Randomized, double-blind	18	Phonematic verbal fluency improved in dual-frequency stimulation (130 + 10 Hz) compared to conventional 130 Hz stimulation; no difference between dual-frequency stimulation and 10 Hz stimulation

Sidiropoulos et al. (2013)[29]	J Neurol.	Non-randomized, non-blinded	45	No significant improvement in speech, gait, axial subscores, and motors scores during LFS (80 Hz) compared to HFS
Stegemöller et al. (2013)[30]	NeuroRehabilitation	Randomized, double-blind	17	HFS reduced UPDRS tremor score in tremor-dominant patients compared to LFS; no differences in non-tremor dominant group between frequencies; no differences in gait, balance, verbal fluency
Vallabhajosula et al. (2015)[31]	Brain Stimulation	Randomized, single-blind	19	Similar effect of LFS (60 Hz) and HFS (> 100 Hz) on postural control and gait; no further improvement with LFS
Xie et al. (2015)[32]	Neurology	Randomized, double-blind	7	LFS (60 Hz) reduced aspiration frequency, perceived swallowing difficulty, and reduced FOG and axial and Parkinsonian symptoms
Xie et al. (2018)[33]	J Neurol Neurosurg Psychiatry.	Randomized, double-blind	11	Reduced aspiration frequency, perceived swallowing difficulty, FOG severity, bradykinesia, and overall axial and motor symptoms except dysphagia during LFS (60 Hz) compared to HFS (130 Hz) with decreased benefits with long-term use
**Variation of pulse width**				
Bouthour et al. (2018)[34]	Mov Disord.	Randomized, double-blind	10	TW widened while PW shortened and CPP was reduced
Dayal et al. (2018)[35]	J Parkinsons Dis.	Non-randomized, single-blind	15	Wider TW with low PW (30 μs) compared to 60 μs, faster timed 10-meter walk, improved speed of gait and perceptual speech scores at 30 μs
Dayal et al. (2020)[36]	Mov Disord.	Randomized, double-blind	16	No difference in Sentence Intelligibility Test (SIT) scores between baseline, 30 μs, and 60 μs; no difference in motor, non-motor, quality of life scores, and TEED; similar adverse event rates; wider TW at 30 μs; post-hoc analysis: higher improvement in SIT scores during 30 μs in patients with a short duration of DBS therapy
Dayal et al. (2020)[2]	Mov Disord.	Randomized, double-blind	32	Increased TW during 30 μs compared to 60 μs; higher speech Intelligibility, lower dyskinesias during 30 μs

Fabbri et al. (2021)[37]	Parkinsonism Relat Disord.	Randomized, blinded rater	7	At high amplitudes, 30 μs improved speech intelligibility without worsening motor function
Moro et al. (2002)[24]	Neurology	Randomized, double-blind	12	Improvement of bradykinesia only significant with 60 μs; no effect of PW variation on tremor; highest tolerated PW had higher rigidity improvement than 60 μs
Mügge et al. (2023)[25]	Neurol Res Pract.	Randomized	23	PW of 90 μs can improve leg lift
Petry-Schmelzer et al. (2022)[38]	J Parkinsons Dis.	Randomized, double-blind	30	No difference between 30 μs and 60 μs regarding on-time, UPDRS-I /-II /-III subscores, PDQ-39 summary index, NMSQ, side effects, TEED, Battery Charge Index; significant lower CPP during 30 μs
Reich et al. (2015)[39]	Ann Clin Transl Neurol.	Randomized, non-blinded	4	TW widened while PW shortened and CPP was reduced
Seger et al. (2021)[40]	J Parkinsons Dis.	Randomized, double-blind	20	60 and 40 μs improved gait velocity compared to off DBS, no difference regarding gait velocity, balance, motor, and non-motor performance; 40% of the patients preferred 40 μs
Steigerwald et al. (2018)[41]	Mov Disord.	Randomized, double-blind	15	Wider TW with a low PW (30 μs) compared to 60 μs, noninferior motor efficacy (UPDRS-III)
**Bipolar stimulation**				
Deli et al. (2011)[42]	Parkinsonism Relat Disord.	Randomized, non-blinded	21	Unipolar stimulation had higher efficacy compared to bipolar; higher rate of SE during unipolar stimulation; bipolar stimulation required higher amplitudes to achieve the same improvement
Hancu et al. (2019)[43]	J Magn Reson Imaging.	Non-randomized, non-blinded	13	Different fMRI activation patterns in monopolar and bipolar configurations; no difference in UPDRS-III scores when amplitude is increased by 30% in the bipolar setting

Soh et al. (2019)[44]	Neuromodulation	Non-randomized, non-blinded	10	Therapeutic threshold, SE threshold and TW is higher during bipolar stimulation compared to cathodic stimulation; higher Battery Consumption Index during bipolar stimulation
**Interleaving stimulation**				
Karl et al. (2020)[45]	Mov Disord.	Randomized, double-blind	25	Interleave-interlink stimulation improved axial and appendicular symptoms compared to conventional HFS

CPP = charge per pulse, DBS = deep brain stimulation, FOG = freezing of gait, HFS = high-frequency stimulation, LFS = low-frequency stimulation, PW = pulse width, SE = side effect, TEED = total electrical energy delivered, TW = therapeutic window, UPDRS = Unified Parkinson’s Disease Rating Scale VFS = variable frequency stimulation.

References

1. Contarino MF, Bour LJ, Verhagen R, et al. Directional steering: A novel approach to deep brain stimulation. *Neurology*. 2014;83(13):1163-1169. doi:10.1212/WNL.0000000000000823
2. Dayal V, De Roquemaurel A, Grover T, et al. Novel Programming Features Help Alleviate Subthalamic Nucleus Stimulation-Induced Side Effects. *Mov Disord*. 2020;35(12):2261-2269. doi:10.1002/mds.28252
3. Dembek TA, Reker P, Visser-Vandewalle V, et al. Directional DBS increases side-effect thresholds—A prospective, double-blind trial. *Mov Disord*. 2017;32(10):1380-1388. doi:10.1002/mds.27093
4. Hurt CP, Kuhman DJ, Moll A, et al. Pointing in the right direction: Greater motor improvements with directional versus circular subthalamic nucleus deep brain stimulation for Parkinson’s disease. *J Park Dis*. 2025;15(1):202-213. doi:10.1177/1877718X241301071
5. Pollo C, Kaelin-Lang A, Oertel MF, et al. Directional deep brain stimulation: an intraoperative double-blind pilot study. *Brain*. 2014;137(7):2015-2026. doi:10.1093/brain/awu102
6. Schnitzler A, Mir P, Brodsky MA, et al. Directional Deep Brain Stimulation for Parkinson’s Disease: Results of an International Crossover Study With Randomized, Double-Blind Primary Endpoint. *Neuromodulation Technol Neural Interface*. 2022;25(6):817-828. doi:10.1111/ner.13407
7. Sekimoto S, Oyama G, Bito K, et al. Three-dimensional gait analysis of the effect of directional steering on gait in patients with Parkinson’s disease. *Parkinsonism Relat Disord*. 2023;114:105770. doi:10.1016/j.parkreldis.2023.105770
8. Steigerwald F, Müller L, Johannes S, Matthies C, Volkmann J. Directional deep brain stimulation of the subthalamic nucleus: A pilot study using a novel neurostimulation device. *Mov Disord*. 2016;31(8):1240-1243. doi:10.1002/mds.26669
9. Hui D, Murgai AA, Gilmore G, Mohideen SI, Parrent AG, Jog MS. Assessing the effect of current steering on the total electrical energy delivered and ambulation in Parkinson’s disease. *Sci Rep*. 2020;10(1):8256. doi:10.1038/s41598-020-64250-7

10. Annic A, Moreau C, Salleron J, et al. Predictive Factors for Improvement of Gait by Low-Frequency Stimulation in Parkinson's Disease. *J Park Dis*. 2014;4(3):413-420. doi:10.3233/JPD-130337
11. Busteed L, García-Sánchez C, Pascual-Sedano B, et al. Impact of Stimulation Frequency on Verbal Fluency Following Bilateral Subthalamic Nucleus Deep Brain Stimulation in Parkinson's Disease. *Arch Clin Neuropsychol*. 2024;40(1):22-32. doi:10.1093/arclin/acae062
12. Cheng Y, Zhao G, Chen L, et al. Effects of subthalamic nucleus deep brain stimulation using different frequency programming paradigms on axial symptoms in advanced Parkinson's disease. *Acta Neurochir (Wien)*. 2024;166(1):124. doi:10.1007/s00701-024-06005-1
13. Conway ZJ, Silburn PA, Perera T, O'Maley K, Cole MH. Low-frequency STN-DBS provides acute gait improvements in Parkinson's disease: a double-blinded randomised cross-over feasibility trial. *J NeuroEngineering Rehabil*. 2021;18(1):125. doi:10.1186/s12984-021-00921-4
14. Dutke J, Gehlenborg J, Heise M, et al. Effects of theta burst stimulation on the Parkinsonian gait disorder and cortical gait-network activity. *J Park Dis*. 2025;15(4):843-857. doi:10.1177/1877718X251320941
15. Fagundes VDC, Rieder CRM, Cruz AND, Beber BC, Portuguez MW. Deep Brain Stimulation Frequency of the Subthalamic Nucleus Affects Phonemic and Action Fluency in Parkinson's Disease. *Park Dis*. 2016;2016:1-9. doi:10.1155/2016/6760243
16. Grover T, Georgiev D, Kalliola R, et al. Effect of Low versus High Frequency Subthalamic Deep Brain Stimulation on Speech Intelligibility and Verbal Fluency in Parkinson's Disease: A Double-Blind Study. *J Park Dis*. 2019;9(1):141-151. doi:10.3233/JPD-181368
17. Herz DM, Blech J, Winter Y, Gonzalez-Escamilla G, Groppa S. Low-Frequency Deep Brain Stimulation in Non-Rapid Eye Movement Sleep Modifies Memory Retention in Parkinson's Disease. *Mov Disord*. 2025;40(2):285-291. doi:10.1002/mds.30064
18. Imbalzano G, Montanaro E, Ledda C, et al. Theta-Gamma Subthalamic Stimulation for Verbal Fluency in Parkinson's Disease: A Randomized, Crossover Trial. *Mov Disord*. 2025;40(6):1189-1194. doi:10.1002/mds.30218
19. Jia F, Shukla AW, Hu W, et al. Variable frequency deep brain stimulation of subthalamic nucleus to improve freezing of gait in Parkinson's disease. *Natl Sci Rev*. 2024;11(6):nwae187. doi: 10.1093/nsr/nwae187.
20. Khoo HM, Kishima H, Hosomi K, et al. Low-frequency subthalamic nucleus stimulation in Parkinson's disease: A randomized clinical trial. *Mov Disord*. 2014;29(2):270-274. doi:10.1002/mds.25810
21. Lee DJ, Drummond NM, Saha U, et al. Acute low frequency dorsal subthalamic nucleus stimulation improves verbal fluency in Parkinson's disease. *Brain Stimulat*. 2021;14(4):754-760. doi:10.1016/j.brs.2021.04.016
22. Moreau C, Defebvre L, Destee A, et al. STN-DBS frequency effects on freezing of gait in advanced Parkinson disease. *Neurology*. 2008;71(2):80-84. doi: 10.1212/01.wnl.0000303972.16279.46.
23. Moreau C, Pennel-Ployart O, Pinto S, et al. Modulation of dysarthropneumophonia by low-frequency STN DBS in advanced Parkinson's disease. *Mov Disord*. 2011;26(4):659-663. doi:10.1002/mds.23538
24. Moro E, Esselink RJA, Xie J, Hommel M, Benabid AL, Pollak P. The impact on Parkinson's disease of electrical parameter settings in STN stimulation. *Neurology*. 2002;59(5):706-713. doi:10.1212/WNL.59.5.706
25. Mügge F, Kleinholdermann U, Heun A, Ollenschläger M, Hannink J, Pedrosa DJ. Subthalamic 85 Hz deep brain stimulation improves walking pace and stride length in Parkinson's disease patients. *Neurol Res Pract*. 2023;5(1):33. doi:10.1186/s42466-023-00263-7
26. Phibbs FT, Arbogast PG, Davis TL. 60-Hz Frequency Effect on Gait in Parkinson's Disease With Subthalamic Nucleus Deep Brain Stimulation. *Neuromodulation Technol Neural Interface*. 2014;17(8):717-720. doi:10.1111/ner.12131
27. Ricchi V, Zibetti M, Angrisano S, et al. Transient effects of 80 Hz stimulation on gait in STN DBS treated PD patients: A 15 months follow-up study. *Brain Stimulat*. 2012;5(3):388-392. doi:10.1016/j.brs.2011.07.001
28. Ricciardi L, Cucinotta F, Pegolo E, et al. Low/High Multi-Frequency Stimulation of the Subthalamic Nucleus Improves Verbal Fluency Maintaining Motor Control in Parkinson's Disease. *Mov Disord*. 2025;40(9):1892-1900. doi:10.1002/mds.30254

29. Sidiropoulos C, Walsh R, Meaney C, Poon YY, Fallis M, Moro E. Low-frequency subthalamic nucleus deep brain stimulation for axial symptoms in advanced Parkinson's disease. *J Neurol*. 2013;260(9):2306-2311. doi:10.1007/s00415-013-6983-2
30. Stegemöller EL, Vallabhajosula S, Haq I, Hwynn N, Hass CJ, Okun MS. Selective use of low frequency stimulation in Parkinson's disease based on absence of tremor. *NeuroRehabilitation*. 2013;33(2):305-312. doi:10.3233/NRE-130960
31. Vallabhajosula S, Haq IU, Hwynn N, et al. Low-frequency Versus High-frequency Subthalamic Nucleus Deep Brain Stimulation on Postural Control and Gait in Parkinson's Disease: A Quantitative Study. *Brain Stimulat*. 2015;8(1):64-75. doi:10.1016/j.brs.2014.10.011
32. Xie T, Vigil J, MacCracken E, et al. Low-frequency stimulation of STN-DBS reduces aspiration and freezing of gait in patients with PD. *Neurology*. 2015;84(4):415-420. doi: 10.1212/WNL.0000000000001184.
33. Xie T, Bloom L, Padmanaban M, et al. Long-term effect of low frequency stimulation of STN on dysphagia, freezing of gait and other motor symptoms in PD. *J Neurol Neurosurg Psychiatry*. 2018;89(9):989-994. doi:10.1136/jnnp-2018-318060
34. Bouthour W, Wegrzyk J, Momjian S, et al. Short pulse width in subthalamic stimulation in Parkinson's disease: a randomized, double-blind study. *Mov Disord*. 2018;33(1):169-173. doi:10.1002/mds.27265
35. Dayal V, Grover T, Limousin P, et al. The Effect of Short Pulse Width Settings on the Therapeutic Window in Subthalamic Nucleus Deep Brain Stimulation for Parkinson's disease. *J Park Dis*. 2018;8(2):273-279. doi:10.3233/JPD-171272
36. Dayal V, Grover T, Tripoliti E, et al. Short Versus Conventional Pulse-Width Deep Brain Stimulation in Parkinson's Disease: A Randomized Crossover Comparison. *Mov Disord*. 2020;35(1):101-108. doi:10.1002/mds.27863
37. Fabbri M, Natale F, Artusi CA, et al. Deep brain stimulation fine-tuning in Parkinson's disease: Short pulse width effect on speech. *Parkinsonism Relat Disord*. 2021;87:130-134. doi:10.1016/j.parkreldis.2021.05.007
38. Petry-Schmelzer JN, Schwarz LM, Jergas H, et al. A Randomized, Double-Blinded Crossover Trial of Short Versus Conventional Pulse Width Subthalamic Deep Brain Stimulation in Parkinson's Disease. *J Park Dis*. 2022;12(5):1497-1505. doi:10.3233/JPD-213119
39. Reich MM, Steigerwald F, Sawalhe AD, et al. Short pulse width widens the therapeutic window of subthalamic neurostimulation. *Ann Clin Transl Neurol*. 2015;2(4):427-432. doi:10.1002/acn3.168
40. Seger A, Gulberti A, Vettorazzi E, et al. Short Pulse and Conventional Deep Brain Stimulation Equally Improve the Parkinsonian Gait Disorder. *J Park Dis*. 2021;11(3):1455-1464. doi:10.3233/JPD-202492
41. Steigerwald F, Timmermann L, Kühn A, et al. Pulse duration settings in subthalamic stimulation for Parkinson's disease. *Mov Disord*. 2018;33(1):165-169. doi:10.1002/mds.27238
42. Deli G, Balas I, Nagy F, et al. Comparison of the efficacy of unipolar and bipolar electrode configuration during subthalamic deep brain stimulation. *Parkinsonism Relat Disord*. 2011;17(1):50-54. doi:10.1016/j.parkreldis.2010.10.012
43. Hancu I, Boutet A, Fiveland E, et al. On the (Non-)equivalency of monopolar and bipolar settings for deep brain stimulation fMRI studies of Parkinson's disease patients. *J Magn Reson Imaging*. 2019;49(6):1736-1749. doi:10.1002/jmri.26321
44. Soh D, Ten Brinke TR, Lozano AM, Fasano A. Therapeutic Window of Deep Brain Stimulation Using Cathodic Monopolar, Bipolar, Semi-Bipolar, and Anodic Stimulation. *Neuromodulation Technol Neural Interface*. 2019;22(4):451-455. doi:10.1111/ner.12957
45. Karl JA, Ouyang B, Goetz S, Metman LV. A Novel DBS Paradigm for Axial Features in Parkinson's Disease: A Randomized Crossover Study. *Mov Disord*. 2020;35(8):1369-1378. doi:10.1002/mds.28048
